# Artificial intelligence and precision medicine: a pilot study predicting optimal ceftaroline dosage for pediatric patients

**DOI:** 10.3389/frai.2025.1702087

**Published:** 2026-01-16

**Authors:** Maria Frasca, Gianluca Gazzaniga, Agnese Graziosi, Valentina De Nicolo, Costantino De Giacomo, Stefano Martinelli, Michele Senatore, Alessandra Romandini, Chiara Moretti, Giulia Angela Carla Pattarino, Alice Proto, Romano Danesi, Francesco Scaglione, Gianluca Vago, Davide La Torre, Arianna Pani

**Affiliations:** 1 Department of Oncology and Hemato-Oncology, University of Milan, Milan, Italy; 2 Department of General Surgery and Surgical Specialty Paride Stefanini, Sapienza University of Rome, Rome, Italy; 3 Department of Medical Biotechnology and Translational Medicine, Postgraduate School of Clinical Pharmacology and Toxicology, University of Milan, Milan, Italy; 4 Pediatric Unit, ASST Grande Ospedale Metropolitano Niguarda, Milan, Italy; 5 Neonatal Intensive Care Unit, ASST Grande Ospedale Metropolitano Niguarda, Milan, Italy; 6 Chemical-Clinical Analyses Unit, ASST Grande Ospedale Metropolitano Niguarda, Milan, Italy; 7 SKEMA Business School, Université Côte d’Azur, Nice, France; 8 Department of Mathematics, University of Bologna, Bologna, Italy

**Keywords:** artificial intelligence, ceftaroline, drug dosing, explainable AI, machine learning, pediatric pharmacokinetics, personalized medicine

## Abstract

**Background:**

Accurate drug dosing in pediatrics is complicated by age-related physiological variability. Standard weight-based dosing may result in either subtherapeutic exposure or toxicity. Machine learning (ML) models can capture complex relationships among clinical variables and support individualized therapy.

**Methods:**

We analyzed clinical and pharmacokinetic data from 20 pediatric patients enrolled in the PUERI study (January 2020–November 2021, ASST Grande Ospedale Metropolitano Niguarda, Milan, Italy). Eight ML models-including linear regression (LR), ridge regression (RR), lasso regression (LaR), Huber regression (HR), random forest (RF), XGBoost, LightGBM, and a neural network (MLP)-were trained to predict ceftaroline doses that would achieve plasma concentrations close to the therapeutic target of 10 mg/L. Model performance was evaluated using mean absolute error (MAE), root mean squared error (RMSE), and the coefficient of determination (R^2^). To ensure interpretability, we applied local interpretable model-agnostic explanations (LIME) to identify the most influential predictors of dose.

**Results:**

MLP (MAE 1.53 mg, R^2^ 0.94) and XGBoost (MAE 2.04 mg, R^2^ 0.89) outperformed linear models. Predicted doses were more frequently aligned with therapeutic concentrations than those clinically administered. Model-based simulated concentrations fell within the therapeutic range in approximately 85% of cases, and the best ML models showed over 90% patient-level clinical. RF, LightGBM and XGBoost achieved the highest clinical alignment, with 94.2, 92.4 and 91.5% of patients reaching therapeutic levels. Renal function markers, such as serum creatinine and azotemia, together with anthropometric parameters including weight, height, and body mass index, were consistently the most influential features.

**Conclusion:**

Advanced ML models can optimize ceftaroline dosing in pediatric patients and outperform traditional dosing strategies. Combining predictive accuracy with interpretability (via LIME) supports clinical trust and may enhance precision antibiotic therapy while reducing the risks of antimicrobial resistance and toxicity.

## 1 Introduction

The correct administration of antibiotics in pediatrics represents a complex challenge for modern pharmacotherapy. Unlike adults, children exhibit significant physiological variability related to age, weight, renal function, and liver maturation, all of which critically influence drug metabolism and elimination (Willems et al., 2021). Standardized dosing based on simple proportional formulas (e.g., mg/kg) is often insufficient to ensure effective therapy personalization, exposing patients to the risk of overdosing, which can lead to adverse effects, or underdosing, with potential therapeutic failure (Anderson and Holford, 2008; Kearns et al., 2003). This challenge is compounded by the growing threat of antimicrobial resistance (AMR). Inappropriate dosing not only risks therapeutic failure but also accelerates the selection and proliferation of drug-resistant bacterial strains, rendering treatments less effective and contributing to one of the most critical global public health threats of this century, with an estimated 10 million deaths per year by 2050 (Murray et al., 2022; Ahmed et al., 2024). In this context, Artificial Intelligence (AI) and Machine Learning (ML) techniques are emerging as innovative tools to address the complexities of personalized medicine, with a particular focus on optimizing drug dosages (Arnold et al., 2025; Smith et al., 2020). By analyzing vast amounts of clinical data and identifying complex patterns among physiological variables, AI models offer a novel approach to individualized pharmacotherapy. This capability enables clinicians to predict optimal antibiotic dosages based on specific patient characteristics, thereby improving treatment efficacy and reducing toxicity risks (Poweleit et al., 2023). Ceftaroline, a fifth-generation cephalosporin, has gained attention for its broad spectrum of action, which includes resistant pathogens like Methicillin-Resistant *Staphylococcus Aureus* (MRSA; Welte et al., 2019). However, its optimal dosage in pediatric patients remains a challenge due to the scarcity of clinical data and significant interindividual variability. While standard doses are outlined in ceftaroline’s summary of product characteristics, ensuring therapeutic drug concentrations while avoiding toxicity is crucial. This critical balance underscores the urgent need for individualized dosing strategies (Ruggiero et al., 2019). This study leverages multiple regression models to predict the optimal ceftaroline dose required to achieve a target therapeutic plasma concentration of 10 mg/L. We compare these AI-predicted doses with those actually administered to identify potential instances of under- or overdosing. We also employ Explainable AI (XAI) technologies, such as Local Interpretable Model-agnostic Explanations (LIME), to enhance model interpretability by highlighting the most influential patient characteristics in dose prediction. This research aims to demonstrate how AI can increase the accuracy of ceftaroline administration, paving the way for safer and more effective treatments for pediatric patients.

## 2 Materials and methods

### 2.1 Study design and data source

This retrospective analysis utilized data from the “Observational, single-center, open-label, sequential, pharmacokinetic and tolerability study of ceftaroline in pediatric patients from 2 to 24 months of age, with suspected or confirmed infection (PUERI).” The PUERI study was conducted between January 2020 and November 2021 at the Pediatric Department of the ASST Grande Ospedale Metropolitano Niguarda (Milan, Italy). The study adhered to the Declaration of Helsinki and Good Clinical Practice guidelines and was approved by the Niguarda Hospital Ethics Committee (protocol no. 189–042019, April 15, 2019). Written informed consent was obtained from parents or legal guardians.

### 2.2 Patient population and data collection

The dataset comprised pharmacological, clinical, and demographic information from 21 pediatric patients; one was excluded due to insufficient data, yielding a final cohort of 20. Ceftaroline therapy was prescribed at physician discretion, with documentation of infusion parameters, dosage, and administration time. Opportunistic plasma sampling used residual specimens from routine clinical procedures. Variables collected are detailed in Supplementary Table S1.

### 2.3 Data preprocessing and feature selection

Prior to model development, the dataset underwent a systematic preprocessing pipeline. First, missing data were handled by mean imputation for each numerical feature. Categorical variables were then converted into a numerical format. To ensure uniform scaling and improve model stability, all selected features were standardized to a mean of 0 and a standard deviation of 1. The features used for training, including anthropometric, biochemical, and derived variables, e.g., BMI and the ratio between target and real plasma concentrations (DV ratio), were selected based on their clinical relevance and availability (Table 1). Time elapsed since the first drug administration (TIME) and Infusion rate (mg/h; RATE) were included as retrospective descriptors of the pharmacokinetic context at sampling and were not used for prospective pre-dose prediction. DV ratio was used only as an exploratory feature during training and had no role in dose optimisation. For patients with multiple treatment cycles, each cycle was treated as a distinct event to ensure accurate representation.

**Table 1:** Features used in the ML models for dose prediction.

Feature	Description
TIME	Time since the start of the infusion (minutes)
RATE	Infusion rate (mL/min)
HT	Patient’s height (cm)
WT	Patient’s body weight (kg)
SCR	Serum creatinine level (mg/dl)
AZOTEMIA	Blood urea nitrogen concentration (mg/dl)
AST	Aspartate Aminotransferase level (U/L)
ALT	Alanine Aminotransferase level (U/L)
Albumin	Blood albumin concentration (g/dl)
Schwartz formula	eGFR estimated using the Schwartz formula
Revised Schwartz equation	eGFR using the revised Schwartz formula
BMI	Body Mass Index calculated from height and weight
DV ratio	Ratio between target and real plasma concentration

### 2.4 Model development and evaluation

The dataset included 20 pediatric patients, contributing a total of 58 pharmacokinetic events. Since several patients had more than one therapeutic cycle, events were treated as independent units. The 80/20 split was performed at the event level (46 training events and 12 independent test events) using a fixed random seed of 42. The independent test set was not used during hyperparameter tuning or model selection. This approach was used to evaluate model generalization and prevent overfitting. We utilized a range of eight ML regression models to predict the personalized optimized ceftaroline dose, including linear regression (LR), ridge regression (RR), Lasso Regression (LaR), Huber regression (HR), Random Forest (RF), XGBoost, LightGBM, and a Neural Network (MLP). Each model was trained with the optimized dose (Optimized AMT) as the dependent variable. Model performance was evaluated on the test set using standard metrics for regression tasks: Root Mean Squared Error (RMSE), Mean Absolute Error (MAE), Coefficient of Determination (R^2^), and Mean Absolute Percentage Error (MAPE; Supplementary file 2).

### 2.5 Model validation and overfitting control

To ensure robustness given the limited sample size, all models were internally validated using a leave-one-out cross-validation (LOOCV) strategy, which maximizes data utilization and provides an unbiased estimate of generalization performance. Additionally, bootstrap resampling (1,000 iterations) was performed to derive 95% confidence intervals for MAE, RMSE and R^2^ and to assess model stability. Regularization techniques (L1/L2 penalties) were applied to linear models, while early stopping and dropout were used for tree-based and neural architectures to minimize overfitting and improve generalization. Hyperparameter tuning for RF, XGBoost, LightGBM and MLP was performed outside the LOOCV framework, using predefined configurations based on best practices in the literature.

### 2.6 Explainable AI analysis and dose comparison

After model training, we calculated a personalized optimized dose for each patient by minimizing the absolute deviation between the predicted drug concentration and the target value of 10 mg/L. This Optimized AMT was then compared to the dose actually administered clinically (AMT) to identify potential under- or overdosing. Finally, to enhance model interpretability, the best-performing models were analyzed using Local Interpretable Model-agnostic Explanations (LIME). This technique was used to identify the most significant clinical variables contributing to the dose predictions, providing insight into the key features driving the model’s recommendations. LIME was implemented using 5,000 perturbed samples, a kernel width of 0.75, quantile-based discretization, and a fixed random seed of 42.

## 3 Results

### 3.1 Model performance and comparison

The ML models demonstrated varying capabilities in predicting the Optimized AMT. A key metric was the R^2^ score, which represents the proportion of variability in the dependent variable explained by the model. The Neural Network showed the highest R^2^ score at 0.94, followed by Random Forest (0.93), XGBoost (0.89), and LightGBM (0.84). The performance of linear models was generally lower, with the Linear Regression achieving an R^2^ of 0.74 and the Ridge, Lasso, and Huber models ranging from 0.35 to 0.54. Prediction accuracy was further assessed using Mean Absolute Error (MAE) and Root Mean Squared Error (RMSE). The Neural Network achieved the lowest MAE (1.53 mg) and RMSE (3.38 mg), indicating high accuracy. XGBoost also performed exceptionally well, with a low MAE of 2.04 mg and RMSE of 4.70 mg. In contrast, linear models showed higher errors, with Ridge Regression having the largest MAE (7.92 mg) and RMSE (9.94 mg). A summary of the performance metrics for all models is presented in Table 2, the whole results are uploaded in Supplementary file 2.

**Table 2:** Performance comparison of all ML models used in the study.

Model	MAE (mg)	RMSE (mg)	R2
Linear Regression	4.34	7.19	0.74
Ridge Regression	7.92	9.94	0.51
Lasso Regression	7.61	9.58	0.54
Huber Regression	7.20	11.41	0.35
Random Forest	4.21	6.10	0.93
XGBoost	2.04	4.70	0.89
LightGBM	2.92	5.59	0.84
Neural Network	1.53	3.38	0.94

### 3.2 Clinical alignment of predicted doses

The clinical utility of the models was evaluated by comparing simulated plasma concentrations derived from predicted doses with the therapeutic range. Clinical alignment was defined as the proportion of patients whose model-predicted dose resulted in simulated plasma concentrations falling within the predefined therapeutic window of 1–10 mg/L. RF achieved the highest clinical alignment (94.2% within range). XGBoost and LGBM performed well (91.5 and 92.4% within range, respectively). In contrast, the MLP and LR showed lower alignment (~49.9%), underscoring that models with slightly higher error may still yield more clinically relevant recommendations. Administered versus predicted doses for all models are provided in Table 3.

**Table 3:** Comparison between clinically administered doses and model-predicted optimized doses (mg) for representative patients.

Dose Admin (mg).	LR	RF	XGBoost	MLP	RR	LaR	HR	LGBM
102.0	102.4	97.4	102.0	102.3	101.9	99.7	100.4	101.6
42.0	46.9	42.2	42.0	41.5	44.2	45.3	43.9	41.9
88.2	89.7	88.3	88.2	87.5	88.1	87.4	87.9	88.1
37.2	38.3	39.3	37.2	37.4	38.0	38.6	37.9	37.3
60.0	67.5	60.4	60.0	60.5	63.2	64.3	63.7	60.4
66.0	63.1	67.1	67.0	65.9	65.8	67.3	65.9	66.7
96.0	83.5	91.4	96.0	95.3	91.9	89.4	90.6	95.7
105.0	106.7	102.6	105.0	106.8	105.5	103.9	104.1	105.6
60.0	67.2	58.1	60.0	60.7	63.4	62.1	63.0	59.8
49.8	56.6	51.7	49.8	47.1	52.3	54.1	53.6	50.9
100.2	93.6	96.2	100.2	100.5	97.8	98.1	99.3	100.7
49.8	43.9	45.0	49.8	49.8	46.7	47.3	47.0	49.5
36.92	36.5	38.1	36.9	37.1	36.8	36.9	37.2	37.0
39.8	35.7	39.0	39.8	40.0	38.5	37.9	38.1	39.7
75.0	72.4	79.5	75.0	75.1	75.6	77.8	76.2	75.8
26.96	28.4	32.1	27.0	27.3	29.1	29.8	29.3	27.8
49.8	48.5	49.2	49.8	49.8	49.1	48.9	49.3	49.9
75.0	74.0	80.4	75.0	75.5	76.0	78.3	76.8	75.3
30.0	29.6	33.0	30.0	30.2	31.4	32.0	31.5	30.6
102.0	107.2	101.7	102.0	100.2	104.5	102.1	102.7	101.9

Predictions from each model are shown alongside the corresponding administered dose. LR, Linear Regression; RF, Random Forest; XGBoost, Extreme Gradient Boosting; MLP, Neural Network; RR, Ridge Regression; LaR, Lasso Regression; HR, Huber Regression; LGBM, Light Gradient Boosting Machine.

### 3.3 Visual representation of model performance

**Figure 1:**
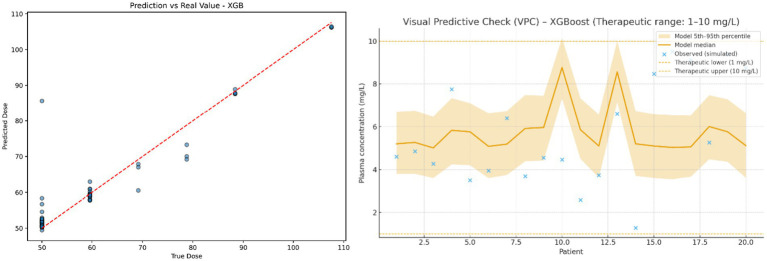
Visual representation of model performance (XGBoost). Left Observed vs. predicted ceftaroline doses. The dashed red line represents the identity line (*y* = *x*); blue points correspond to individual patients. Right Distributional visual predictive check (VPC). The shaded yellow area represents the 5th–95th percentile prediction interval derived from 1,000 bootstrap simulations, and the orange line indicates the model median. Blue crosses show the observed plasma concentrations. The dashed red lines delimit the therapeutic range (1–10 mg/L). This VPC illustrates the agreement between the observed data and the predictive distribution generated by the model, rather than a point-by-point fit.

To clearly assess the accuracy and clinical coherence of the best-performing model (XGBoost), two graphical analyses were generated and are presented in Figure 1. Figure 1A shows the relationship between the predicted and observed ceftaroline doses, highlighting a strong correspondence between the two values across patients. This result confirms the model’s ability to capture individual variability in dosing requirements. Figure 1B presents the Visual Predictive Check (VPC), which compares the observed plasma concentrations with those simulated using the optimized doses predicted by the XGBoost model (Supplementary file 2; Ratain et al., 2003). Approximately 85% of the simulated plasma concentrations fall within the therapeutic range of 1–10 mg/L. Although some variability is still present, which is expected in a small pediatric cohort, the overall consistency of the results supports the validity of XGBoost as a robust proof-of-concept tool for individualized dose optimization.

**Figure 2:**
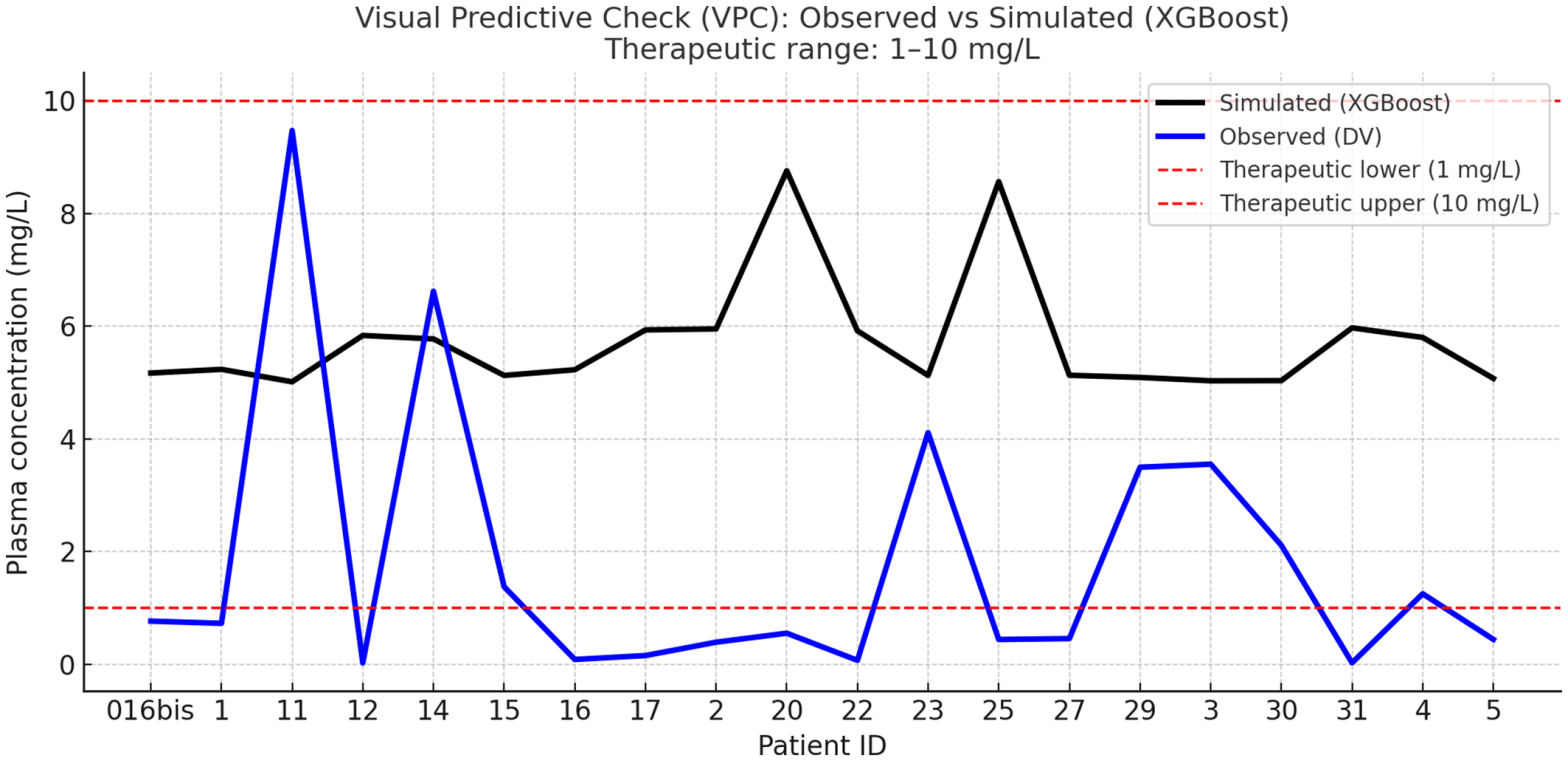
Combined visual predictive check (XGBoost). Blue line: median observed plasma concentrations (DV) per patient. Black line: median simulated concentrations obtained from model-predicted optimized doses. Red dashed lines: therapeutic range limits (1–10 mg/L). This combined VPC compares the observed and model-simulated concentration trends across patients. The divergence between curves reflects the difference between empirical dosing and model-optimized dosing, confirming the model’s ability to reproduce the overall distributional pattern while reducing inter-patient variability.

To further validate the model’s predictive consistency, an additional VPC was performed by comparing the observed plasma concentrations with those simulated using the optimized doses predicted by the XGBoost model. As shown in Figure 2, the observed and simulated concentration curves exhibited a consistent overall trend across patients, confirming that the model accurately captured the shape and magnitude of the plasma concentration distribution. Approximately 85% of simulated concentrations and 37.5% of observed concentrations were within the therapeutic range of 1–10 mg/L, supporting the model’s ability to enhance dosing precision and minimize the risk of sub- or supra-therapeutic exposure. It is important to distinguish patient-level clinical alignment (91–94% depending on the model) from the Visual Predictive Check, where approximately 85% of simulated concentrations fell within the therapeutic window. Moreover, the VPCs demonstrated that the model preserved patient-specific exposure patterns while recentering the simulated concentrations around the therapeutic target (≈10 mg/L), indicating that the XGBoost-based optimization improved the dose homogeneity without diminishing interindividual variability.

### 3.4 Correlation between clinical variables and dose

**Figure 3:**
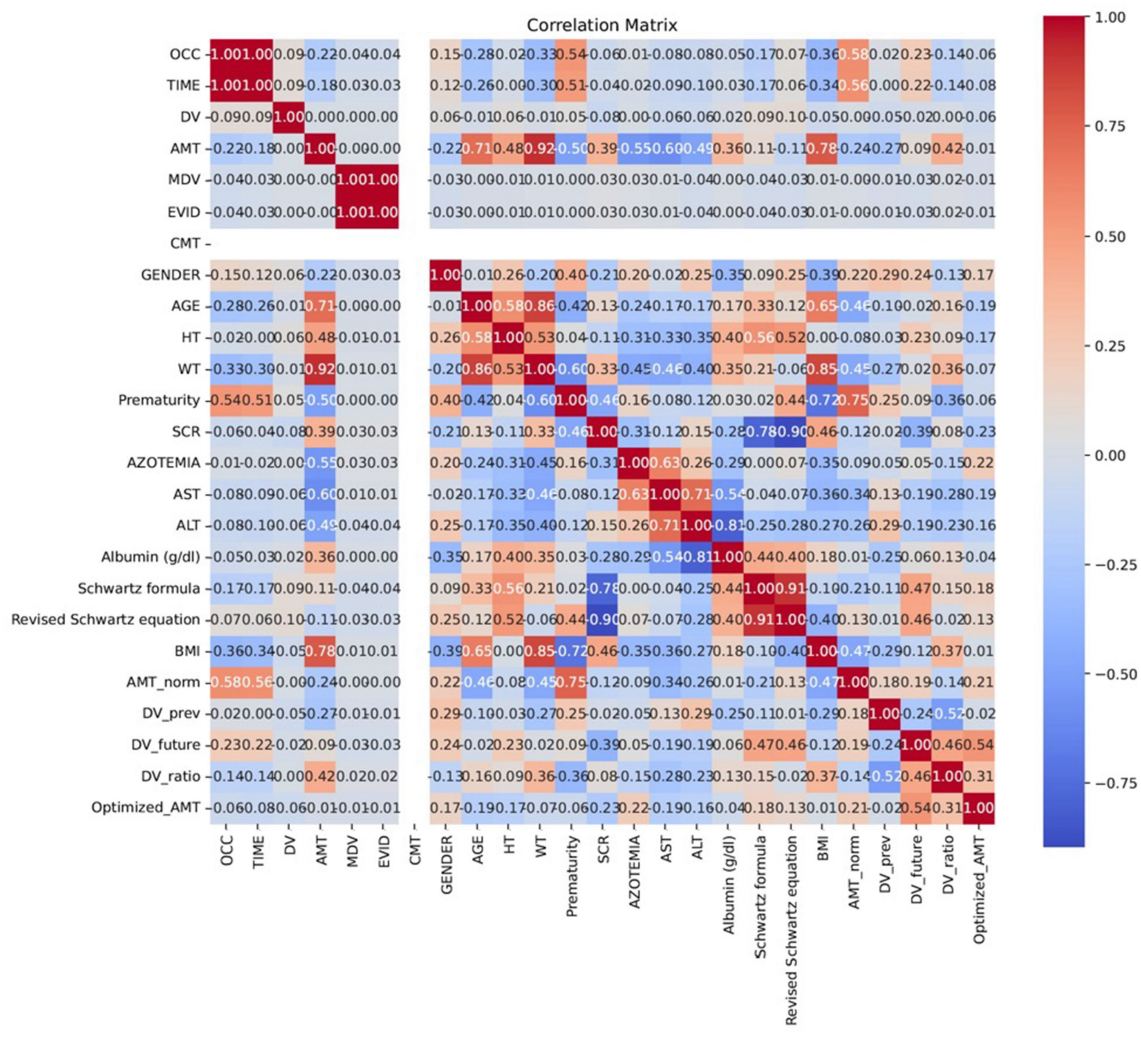
Correlation matrix of clinical variables. The figure highlights the linear relationships between key predictors, such as WT, HT, SCR, and the AMT. Variables like albumin, AST, and ALT show weaker correlations, indicating a less direct influence on the target variable.

A correlation matrix was used to analyze the linear relationships between the clinical variables and the target dose. This analysis was crucial for identifying the variables with the greatest impact on dose prediction and for understanding the underlying relationships in the data. As expected, physiological parameters like WT (r = 0.82), HT (r = 0.76), and SCR (r = 0.68) showed the strongest positive correlations with the dose. This reflects their direct influence on drug pharmacokinetics. Other variables, such as albumin and liver function parameters (AST, ALT), showed weaker correlations, suggesting a less significant direct impact on dose prediction. Figure 3 displays the complete correlation matrix, highlighting the relationships among all variables.

### 3.5 Explainable AI with LIME

**Figure 4:**
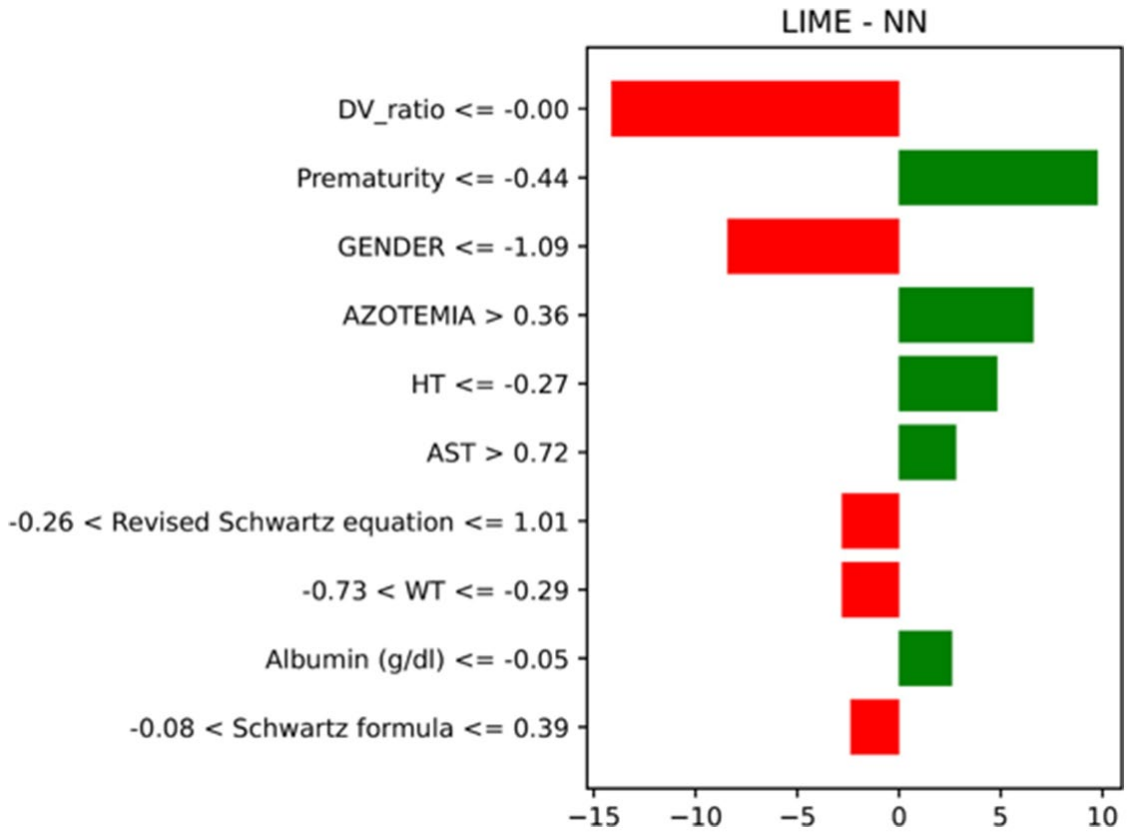
LIME explanation for a representative patient generated by MLP. The graph illustrates the contribution of individual clinical variables to the predicted dose. Positive contributions indicate variables that increased the predicted dose, while negative contributions represent variables that reduced the prediction.

In this study, one of the main objectives was to ensure the interpretability of the predictions made by the ML models. LIME creates a simpler model to explain the behaviour of a complex model for a single prediction, highlighting the features that contributed most to a specific outcome. This approach proved invaluable for local interpretation at an individual patient level. LIME analyses consistently identified WT, HT, SCR, and azotemia as the most influential features across models, with additional contributions from albumin and liver enzymes in some cases. The five most important predictors across models are summarized in Table 4. An illustrative LIME example for MLP is shown in Figure 4, which highlights how specific clinical variables influenced the Optimized AMT for an individual patient. The example shown corresponds to the best-performing model, the Neural Network, selected for its superior predictive accuracy and robustness.

**Table 4:** The top five features contributing to dose predictions across ML models.

Model	Top 5 Features
Linear Regression	Weight (WT), Height (HT), SCR, Azotemia, Age
Ridge Regression	Weight (WT), Height (HT), SCR, Azotemia, Age
Lasso Regression	Weight (WT), Height (HT), SCR, Azotemia, Albumin
Huber Regression	Weight (WT), Height (HT), SCR, Azotemia, Age
Random Forest	Weight (WT), Height (HT), SCR, Azotemia, Albumin
XGBoost	Weight (WT), Height (HT), SCR, Azotemia, AST
LightGBM	Weight (WT), Height (HT), SCR, Azotemia, AST
Neural Network	Weight (WT), Height (HT), SCR, Azotemia, AST

## 4 Discussion

This study presents a comprehensive evaluation of various ML models for predicting an optimized Ceftaroline dose, focusing on both predictive accuracy and clinical utility. Our results establish a clear hierarchy of performance, with advanced models like the MLP and XGBoost demonstrating excellent predictive capability. Specifically, the MLP achieved a MAE of 1.53 mg and an R^2^ of 0.94, while XGBoost performed similarly with an MAE of 2.04 mg and an R^2^ of 0.89. The performance of these models significantly surpassed that of traditional linear methods. RR and LaR, for instance, yielded notably lower R^2^ scores (0.51 and 0.54, respectively) and higher MAEs (7.92 mg and 7.61 mg), confirming their limitations in capturing the complex, non-linear relationships inherent in pharmacokinetic data. Similarly, HR, while more stable with outliers, did not match the performance of the tree-based and neural models, achieving a MAE of 7.20 mg and an R^2^ of 0.35. The efficacy of gradient boosting was further underscored by LightGBM, which delivered performance metrics close to those of XGBoost (MAE of 2.92 mg and R^2^ of 0.84). Beyond mathematical accuracy, our analysis revealed a crucial finding regarding the clinical utility of the models. While the MLP provided the most precise predictions from a statistical standpoint, ensemble models like RF and XGBoost showed superior performance in producing clinically meaningful recommendations. Specifically, XGBoost, RF and LGBM predicted doses that resulted in therapeutic plasma concentrations for 91.5, 94.2 and 92.4% of patients, respectively, while the MLP and LR achieved lower alignment (≈49.9%). This discrepancy highlights a critical point for clinical decision support systems: a low mathematical error does not always equate to high clinical relevance. The high accuracy of the MLP may stem from its ability to model complex, non-linear patterns that lead to highly precise yet potentially out-of-range dose predictions. Conversely, the ensemble models, despite slightly lower accuracy, consistently recommended doses that fall within a clinically safe and effective therapeutic window, making them more valuable in a real-world setting. This suggests that, for dose optimization, models that prioritize a high percentage of clinically aligned predictions may be more valuable than those focused solely on minimizing mathematical error. The interpretability of these models was a cornerstone of our study, achieved through both correlation analysis and the application of LIME. Correlation analysis validated the strong influence of key physiological parameters, such as WT, HT, and SCR, which aligns with established pharmacokinetic principles for Ceftaroline. Notably, we observed a strong correlation between WT and HT (r = 0.89) and between SCR and blood urea nitrogen (r = 0.72). The application of LIME to “black-box” models, such as the MLP, provided critical, per-patient explanations for predictions. This is vital for clinical adoption as it allows physicians to trust and understand the reasoning behind a recommended dose, ensuring consistency with their expertise. Our analysis of feature importance confirmed the central role of renal parameters like SCR and azotemia in predicting dosage, which is consistent with the primary route of Ceftaroline elimination (Zhou et al., 2021). However, given the pharmacokinetic vulnerability of neonatal and especially preterm patients, all model-derived optimized doses in this study were considered strictly exploratory and were not used for clinical decision-making. Moreover, the uneven distribution of renal function markers in this small cohort may bias model behaviour and dose suggestions, and this potential source of error cannot be robustly quantified without larger prospective datasets. Conversely, the appearance of features such as AST and ALT among locally important variables in LIME explanations is likely influenced by multicollinearity and small-sample variability, and was therefore not interpreted as indicating a direct mechanistic role in dose–concentration prediction. Interestingly, our analysis also uncovered subtle sex-based differences, with males tending to require slightly higher doses. While modest, this finding suggests that sex-specific physiological factors, such as differences in body composition or renal function, may warrant further investigation to enhance the precision and equity of future dosing models. Traditional population pharmacokinetic (popPK) models remain the cornerstone of dose optimization, offering valuable mechanistic insights into how drugs are absorbed, distributed, metabolized, and eliminated. However, these models often require predefined structural assumptions and may not easily capture the complex, non-linear relationships that exist among clinical variables in real-world settings. In this work, we propose a complementary, data-driven perspective: the use of ML to model such variability directly from clinical data, without relying on strict compartmental structures. Rather than replacing popPK approaches, our method can enhance them, helping clinicians explore patient-specific factors and identify subtle interactions that traditional frameworks might overlook especially for peculiar population such as infants. The inclusion of explainability tools such as LIME further bridges the gap between computational modeling and clinical reasoning, allowing predictions to be interpreted in a way that aligns with medical decision-making.

## 5 Significance of the study and limitations

This study, although based on a limited pediatric cohort (n = 20), serves as a proof of concept for the application of ML in personalized dose optimization. The small sample size reflects the well-known challenges of conducting pharmacokinetic research in neonates and young infants but nonetheless provides valuable insights in this difficult-to-study population. The strong performance of advanced models warrants validation in larger, multicenter cohorts. Despite the limited sample size, the risk of model overfitting was carefully mitigated through the use of LOOCV and bootstrap-based validation, combined with regularization and early stopping strategies. The convergence and performance metrics remained stable across validation iterations, supporting the reliability of the findings within the intended proof-of-concept framework. Nevertheless, larger multicentric datasets and external validation will be essential to confirm model generalizability and clinical applicability. Future research will also explore the use of synthetic data generation to expand training variability and further enhance model robustness. Formal hypothesis testing for pairwise model comparison was not performed because paired statistical tests are underpowered and unstable in datasets of this size; therefore, model performance is reported descriptively using LOOCV metrics and bootstrap confidence intervals.

## 6 Conclusion

Advanced ML models, particularly XGBoost, RF, and MLP, improve the prediction of ceftaroline dosing in pediatric patients. While MLP achieved the greatest statistical accuracy, ensemble models demonstrated superior clinical alignment, underscoring the importance of balancing precision with therapeutic applicability. The successful application of LIME and correlation analysis further validates that ML models can be both powerful and interpretable, a prerequisite for their seamless integration into clinical practice. The analysis of clinical parameters reaffirmed the predominant role of renal function and anthropometric characteristics in dose determination, which is consistent with known pharmacological principles. These findings advocate for AI-driven dose optimization to advance personalized pediatric pharmacotherapy and contribute to mitigating AMR (Branda and Scarpa, 2024).

## Data Availability

The raw data supporting the conclusions of this article will be made available by the authors, without undue reservation.

## Glossary

AI: Artificial Intelligence
AMR: Antimicrobial Resistance
AMT: Amount administered
ALT: Alanine Aminotransferase
AST: Aspartate Aminotransferase
BMI: Body Mass Index
DV ratio: Ratio between target and real plasma concentration
eGFR: Estimated Glomerular Filtration Rate
HR: Huber Regression
LaR: Lasso Regression
LGBM: Light Gradient Boosting Machine
LIME: Local Interpretable Model-agnostic Explanations
LR: Linear Regression
MAE: Mean Absolute Error
MAPE: Mean Absolute Percentage Error
MLP: Neural Network
PK: Pharmacokinetics
RF: Random Forest
RMSE: Root Mean Squared Error
RR: Ridge Regression
SCR: Serum Creatinine
WT: Weight
HT: Height
XAI: Explainable AI
XGBoost: Extreme Gradient Boosting
