# Supplementary material for: Artificial intelligence and precision medicine: a pilot study predicting optimal ceftaroline dosage for pediatric patients
Source: Front Artif Intell. 2026 Jan 16;8:1702087. doi: 10.3389/frai.2025.1702087 (PMC12856755; doi:10.3389/frai.2025.1702087)
Supplement: Supplementary file 1 [file Data_Sheet_1.docx]

Supplementary file 2

**MODELS USED FOR THE ANALYSIS**

**Linear Regression Model**

The linear regression model predicts the target variable *y* as:

*y* = *β*_0_ + *β*_1_*x*_1_ + *β*_2_*x*_2_ + ··· + *β_p_x_p_* + *ϵ*

where:

- *β*_0_: Intercept term,
- *β*_1_,*β*_2_,*...*,*β_p_*: Coefficients for the *p* features *x*_1_,*x*_2_,*...*,*x_p_*,
- *ϵ*: Error term, assumed to be normally distributed with a mean of 0.

To determine the coefficients *β*, the model minimizes the residual sum of squares (*RSS*), defined as:

*n*

*RSS* = ^X^(*y_i_* − *y*ˆ*_i_*)^2^

*i*=1

where *y_i_* is the observed value, ˆ*y_i_* is the predicted value, and *n* is the total number of observations [10].

Parameters used in this model are:

- **Target Variable (***y***):** The Optimized AMT, calculated to achieve a plasma concentration close to 10 mg/L.
- **Features (***x***):** The model used 13 clinical variables, including BMI and DV ratio, as predictors (see Table I).
- **Normalization:** Before fitting the model, all features were standardized to have a mean of 0 and a standard deviation of 1. This ensures that the coefficients *β* reflect the relative importance of each feature without being influenced by differences in scale.
- **Optimization:** The coefficients were estimated using the ordinary least squares (OLS) method, which guarantees the best linear unbiased estimators under the model’s assumptions.

Linear regression relies on several key assumptions:

1. **Linearity:** A linear relationship exists between the predictors and the target.
2. **Independence:** The residuals are independent of each other.
3. **Homoscedasticity:** The variance of residuals is constant across all levels of the predictors.
4. **Normality of Errors:** The residuals are normally distributed.

The strength of the linear regression model lies in its interpretability. Each coefficient quantifies the effect of a unit change in a feature on the target variable, holding all other variables constant.

**Ridge Regression Model**

Ridge regression is a regularized version of linear regression that introduces an *L*_2_ penalty to the loss function [11]. This penalty term helps control the size of the regression coefficients, which is especially useful in the presence of multicollinearity among features.

The objective function minimized by Ridge regression is:

*n p*

minX(*yi* − *y*ˆ*i*)2 + *λ*X*βj*2 *β*

*i*=1 *j*=1

where *λ* is the regularization strength.

In our implementation, *λ* = 10.0 was used as the penalty coefficient. The model was trained on the same 13 standardized features used in all other regressors. Ridge regression helped prevent overfitting while maintaining all predictors in the model.

**Lasso Regression Model**

Lasso regression is another linear model with regularization, this time based on the *L*_1_ norm. It encourages sparsity in the coefficient vector by shrinking some coefficients to exactly zero, effectively performing feature selection [12].

The objective function minimized is:

*n p*

min^X^(*y_i_* − *y*ˆ*_i_*)^2^ + *λ*^X^|*β_j_*| *β*

*i*=1 *j*=1

In our experiments, we used *λ* = 0.05, a value chosen to balance bias and variance. The Lasso model was helpful for identifying the most influential clinical variables in dose prediction.

**Huber Regression Model**

Huber regression is a robust linear regression technique that combines squared loss (used in OLS) with absolute loss, reducing the influence of outliers in the dataset [13]. The Huber loss function *L_δ_* is defined as:


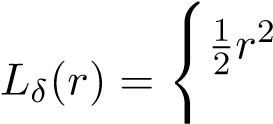
 for |*r*| ≤ *δ*


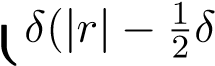
) for |*r*| *> δ*

We used *δ* = 1.2, which allowed the model to be robust to extreme values in DV or AMT.

This model performed competitively on noisy or highly variable clinical inputs.

**LightGBM Regressor Model**

LightGBM (Light Gradient Boosting Machine) is a highly efficient gradient boosting framework that constructs trees using a leaf-wise growth strategy, often leading to faster training and better accuracy on large datasets [14]. In our implementation, the following parameters were used:

- **n estimators:** 100
- **max depth:** 4
- **learning rate:** 0.05
- **subsample:** 0.8
- **colsample bytree:** 0.8
- **random state:** 42

**Random Forest Regressor Model**

The second machine learning model employed in this study was the **Random Forest Regressor**, a powerful and flexible ensemble learning method. This model combines the predictions of multiple decision trees, each trained on different subsets of the data, to enhance accuracy and reduce overfitting [15]. By averaging the predictions from individual trees, Random Forest proves particularly effective for datasets with complex and non-linear relationships. In this study, the Random Forest model was used for two distinct purposes: (1) to estimate DV future values based on the actual or predicted dose and clinical features; and (2) as one of the regression models used to predict Optimized AMT.

The Random Forest Regressor was configured with the following parameters to optimize its performance:

- **Number of Trees:** A total of *n* = 100 decision trees were used in the ensemble. This value represents a balance between model accuracy and computational efficiency, ensuring sufficient diversity among trees while avoiding excessive training times.
- **Maximum Depth:** A maximum depth of 10 was selected to control tree complexity and reduce overfitting.
- **Minimum Samples Split:** The minimum number of samples required to split a node was set to 2.
- **Minimum Samples Leaf:** Set to 4 to ensure greater generalization and reduce noise.
- **Random State:** A fixed random seed (42) was used to ensure reproducibility.
- **Bootstrap Sampling:** Enabled (default), allowing each tree to be trained on a randomly sampled subset of the data with replacement.
- **Maximum Features:** Set to the square root of the number of features (^√^*p*).

Feature importances generated by this model were also used to evaluate the relative influence of each clinical variable on the dosing decision. These parameter settings were carefully selected to fully leverage the power of the Random Forest algorithm, ensuring a robust and reliable model capable of handling the complex relationships inherent in the dataset.

**XGBoost Regressor Model**

The third machine learning model utilized in this study was **XGBoost (Extreme Gradient Boosting)**, a sophisticated ensemble method that builds decision trees sequentially to improve prediction accuracy. Each tree corrects the errors of its predecessor by minimizing a specified loss function, allowing XGBoost to effectively capture non-linear relationships and complex interactions within the data [16]. Its iterative approach, combined with computational optimizations, makes it highly efficient for large-scale datasets.

The XGBoost Regressor was configured with the following parameters:

- **Number of Trees:** 100, chosen to minimize residual errors without overfitting or excessive computation.
- **Learning Rate:** Set to 0.05 to ensure gradual learning and better generalization.
- **Maximum Depth:** Limited to 4 levels to prevent overfitting and reduce model variance.
- **Subsample Ratio:** 0.8, randomly sampling 80% of the training data for each tree to enhance generalization.
- **Column Subsampling:** colsample bytree set to 0.8, to reduce correlation between trees.
- **Regularization (***λ***):** A value of 1 for L2 regularization, preventing large weights and overfitting.
- **Objective Function:** *reg:squarederror*, minimizing the mean squared error (MSE) between predicted and actual values.
- **Random Seed:** 42, ensuring reproducibility.

XGBoost was particularly effective in capturing complex, non-linear patterns between features and dose, while maintaining robustness to noise and overfitting. Among the tree-based models tested, it consistently showed strong performance across all metrics.

**Neural Network Model**

The final machine learning model employed in this study was a **Neural Network**, designed to model complex, non-linear relationships between the input features and the target variable. Neural networks consist of interconnected layers of neurons, where each neuron processes input data and passes the result to the next layer through a weighted combination and an activation function [17]. This architecture enables the network to learn intricate patterns and dependencies within the data, making it highly effective for regression tasks.

A Multilayer Perceptron (MLP) regressor was implemented using the sklearn.neural network. MLPRegressor class. The network was optimized to predict the Optimized AMT based on 13 standardized clinical features.

The neural network architecture and training details were as follows:

- **Input Layer:** 13 neurons, corresponding to all numeric features used in the model, including derived variables such as BMI and DV ratio.
- **Hidden Layers:** Two hidden layers with 50 and 25 neurons, respectively, both using ReLU activation.
- **Output Layer:** A single neuron with a linear activation function for continuous predictions.
- **Optimization:** Trained using Adam with an initial learning rate of 0.01, as specified in the model configuration.
- **Regularization:** L2 regularization (*λ* = 0.001) and dropout (rate = 0.2) were applied to prevent overfitting.
- **Training Configuration:** The network was trained for 500 epochs with a batch size of

32.

The neural network demonstrated the capacity to capture subtle and complex dependencies across multiple clinical variables, though at the cost of lower interpretability compared to treebased models.

**Model Evaluation**

The metrics employed for evaluating the models include RMSE, the MAE, the *R*^2^, and MAPE. The RMSE measures the average squared difference between observed and predicted values. By squaring the differences, this metric emphasizes larger errors, making it particularly suitable for scenarios where such deviations are critical.

RMSE =
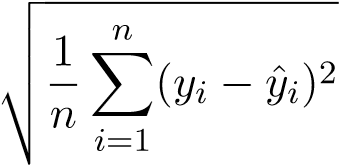


Where:

- *y_i_*: Observed (true) value at position *i*.
- *y*ˆ*_i_*: Predicted value by the model at position *i*.
- *n*: Total number of observations.
- (*y_i_* − *y*ˆ*_i_*)^2^: Squared difference between observed and predicted values.

Finally, the square root is applied to return the result to the original scale of the data. Lower RMSE values signify higher accuracy, although the metric is sensitive to outliers.

The MAE represents the average absolute difference between observed and predicted values. Unlike RMSE, it is less sensitive to outliers, as it does not square the errors.

MAE =
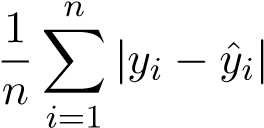


Where:

- *y_i_*: Observed value at position *i*.
- *y*ˆ*_i_*: Predicted value by the model.
- |*y_i_* − *y*ˆ*_i_*|: Absolute error for each observation.
- *n*: Total number of observations.

This metric provides a straightforward measure of average prediction error, expressed in the same units as the data, without giving extra weight to large deviations.

The *R*^2^ quantifies the proportion of variability in the observed data that is captured by the model. Values closer to 1 indicate better performance.


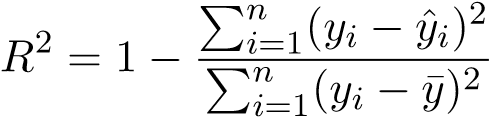


Where:

- *y_i_*: Observed value at position *i*.
- *y*ˆ*_i_*: Predicted value by the model.
- *y*¯: Mean of observed values, computed as ¯
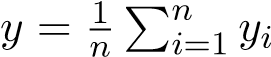
 .
-
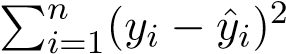
: Sum of squared prediction errors.
-
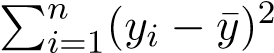
: Total variability in the data.

An *R*^2^ value of 1 represents perfect prediction, while values closer to 0 indicate poor performance. Negative values suggest that the model performs worse than using the mean as a predictor.

The MAPE expresses the average prediction error as a percentage of observed values, making it useful for comparing datasets of different scales.

MAPE =
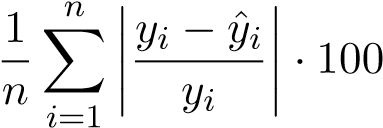


Where:

- *y_i_*: Observed value.
- *y*ˆ*_i_*: Predicted value.
-
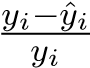
: Relative error for each observation.
- *n*: Total number of observations.

Lower MAPE values indicate higher accuracy in relative terms. However, the metric can be skewed when observed values approach zero, amplifying relative errors.

**Plasma Concentrations Predictions**

Because plasma concentrations were not available, as a predictive model was used to identify the optimal dose to be administered, the analysis was conducted retrospectively with respect to the actual drug administration. Simulated values were therefore generated in line with the best model’s predictive accuracy (XGBoost, R² ≈ 0.89, MAE ≈ 2.04 mg), in order to visualize how the model would behave under realistic pharmacokinetic variability.

The simulated concentrations were calculated using the following simplified equations:

$$C_{\left\{ obs,i \right\}}= \alpha\times{TrueDose}_{i} + \varepsilon_{i}, con \varepsilon_{i} \sim N(0,1.5)$$

$$C_{pred, i} = \alpha\times{Pred(XGB)}_{i} + \varepsilon\{i\}$$

where α = 0.1 was used as a scaling factor to convert doses, expressed in milligrams, into approximate concentrations.

This simplified linear formulation is not intended to describe the full pharmacokinetic dynamics, but rather to provide an empirical link between administered or predicted doses and the corresponding plasma concentrations. In this context, α acts as a conversion factor between dose and concentration, while εi represents the residual biological variability typically arising from patient-specific factors such as metabolism, age, or sampling time. This approach preserves the expected proportionality between dose and exposure, while introducing a realistic degree of random fluctuation. In essence, it serves as a mathematical bridge that allows visualization of how the model’s predictions translate into plausible plasma concentration distributions, without assuming a detailed compartmental pharmacokinetic structure.

Supplementary Figure 1. Comparison between observed and predicted values for the a) Linear Regression model, b) Ridge regression model, c) Lasso regression model, d) Huber regression model, e) LightGBM model, f) Random Forest model, g) XGBoost model, h) Neural Network model. The red diagonal line represents perfect prediction, with points closely aligned indicating minimal bias and strong accuracy.
