# Supplementary material for: Artificial intelligence and precision medicine: a pilot study predicting optimal ceftaroline dosage for pediatric patients
Source: Front Artif Intell. 2026 Jan 16;8:1702087. doi: 10.3389/frai.2025.1702087 (PMC12856755; doi:10.3389/frai.2025.1702087)
Supplement: Supplementary file 2 [file Table_1.docx]

# Supplementary Table S1. Dataset structure and clinical variables collected.

This table summarizes the dataset structure and clinical variables collected, including anthropometric, biochemical, and pharmacological parameters.

| **Variable** | Description |
| --- | --- |
| ID | Unique identifier for each patient. Patients with treatments in separate periods are identified individually. |
| OCC | Progressive treatment day, reset to 1 for each new patient. |
| TIME | Time elapsed since the first drug administration, expressed in decimal hours (e.g., 13.50 = 13 hours and 30 minutes). |
| **Pharmacokinetic Data** |  |
| DV | Plasma concentration of ceftaroline (mg/L). Numeric values indicate measurements, while a dot signifies an administration event. |
| AMT | Amount of ceftaroline administered (mg). Not present when DV is numeric. |
| RATE | Infusion rate (mg/h), calculated by dividing the administered amount (AMT) by the infusion duration (DURATION). |
| DURATION | Infusion duration in hours (e.g., 1 hour = 1, half an hour = 0.5). |
| MDV and EVID | Variables commonly used in pharmacometrics to distinguish between administrations (1) and measurements (0). |
| CMT | Pharmacokinetic compartment (always 1, corresponding to a onecompartment model, as both drug administration and plasma sampling occur in the blood). |
| **Demographic and Clinical Covariates** |  |
| GENDER | Gender (1 = Female, 0 = Male). |
| AGE | Patient age in days. |
| HT | Patient height (cm), used in renal function calculation formulas. |
| WT | Patient weight (kg). |
| Prematurity | Indicator of prematurity (1 = Premature, born before the 37th week; 0 = Full term). |
| **Clinical and Laboratory Parameters** |  |
| AZOTEMIA | Serum azotemia (mg/dL), another parameter for assessing renal function. |
| SCR | Serum creatinine (mg/dL), a key index of renal function |
| AST and ALT | Aspartate Aminotransferase and Alanine Aminotransferase (U/L), markers of hepatic necrosis. |
| Albumin | Plasma albumin (g/dL), essential for drug binding and transport in the blood. |
| **Calculation Formulas** |  |
| Schwartz formula | Standard formula for estimating renal function in children, based on creatinine, height, and prematurity. |
| Revised Schwartz equation | Updated variant of the previous formula with revised parameters. |
